# Gender Differences in the Prevalence of Mental Health, Psychological Distress and Psychotropic Medication Consumption in Spain: A Nationwide Population-Based Study

**DOI:** 10.3390/ijerph18126350

**Published:** 2021-06-11

**Authors:** Clara Maestre-Miquel, Ana López-de-Andrés, Zichen Ji, Javier de Miguel-Diez, Arturo Brocate, Sara Sanz-Rojo, Antonio López-Farre, David Carabantes-Alarcon, Rodrigo Jiménez-García, José J. Zamorano-León

**Affiliations:** 1 School of Health Sciences, Universidad de Castilla la Mancha, 45600 Talavera de la Reina, Spain; Clara.Maestre@uclm.es; 2 Department of Public Health & Maternal and Child Health, Faculty of Medicine, Universidad Complutense de Madrid, 28040 Madrid, Spain; ssanz01@ucm.es (S.S.-R.); dcaraban@ucm.es (D.C.-A.); rodrijim@ucm.es (R.J.-G.); josejzam@ucm.es (J.J.Z.-L.); 3 Respiratory Care Department, Hospital General Universitario Gregorio Marañón, Instituto de Investigación Sanitaria Gregorio Marañón (IiSGM), Universidad Complutense de Madrid, 28040 Madrid, Spain; jizich72@gmail.com (Z.J.); javier.miguel@salud.madrid.org (J.d.M.-D.); 4 Sport Science School, Universidad Castilla de la Mancha, 45071 Toledo, Spain; arturobrocate@gmail.com; 5 Department of Medicine, Faculty of Medicine, Universidad Complutense de Madrid, 28040 Madrid, Spain; antonio.lopez.farre@med.ucm.es

**Keywords:** mental health, psychological distress, psychotropic medication, sex differences, prevalence

## Abstract

Background: To assess gender differences in the prevalence of self-reported mental disorders, psychological distress and psychotropic drug consumption, and to identify sociodemographic and health-related variables associated with these conditions in the male and female population (aged ≥ 18 years). Methods: A cross-sectional study was carried on 22,141 subjects aged 18 and over, using data from the Spanish National Health Interview Survey 2017. Results: We found an overall prevalence of mental disorders, psychological distress and psychotropic drug consumption of 13.8%, 18.3% and 13.9%, respectively. After multivariable adjustment, women showed significantly increased probabilities of 1.74-fold for mental disorders, 1.26-fold for psychological distress and 1.26-fold for psychotropic drug consumption compared to men. Variables such as gender, age, nationality, marital status, educational level, self-rated health, the presence of different chronic disorders, alcohol consumption and smoking habit were independently associated with mental disorders, psychological distress and psychotropic drug consumption. Several variables showed a differential effect on mental health status and psychotropic drug consumption according to gender. Conclusions: Women suffer from mental disorders, experience psychological distress and consume psychotropic drugs significantly more than men in Spain. Possible explanations for these results may be related to differences in emotional processing, willingness to report diseases and even intrinsic biological traits. Screening for mental health status and psychotropic drug consumption should be considered, particularly in Spanish women, younger adults and individuals who are not married, are obese, have poor self-rated health, suffer from chronic diseases or have a smoking habit.

## 1. Introduction

Mental health is considered to be a crucial marker of the overall wellbeing of individuals, societies and countries [1]. Regrettably, mental health problems are currently among the ten leading causes of disability in both developed and developing countries. Indeed, the World Health Organization has estimated that at least 10% of the world’s population have been diagnosed with different types of mental disorders, the most common being anxiety and depression [1]. In particular, depression is ranked third in the global burden of disease and is projected to rank first in 2030 [2].

The European Study of the Epidemiology of Mental Disorders (ESEMeD) is the widest European study published on mental health [3]. This study reveals the high prevalence of mental disorders in Europe, with up to 25.9% of Europeans having been diagnosed with a mental disorder at some point in their life [4]. Historically, Spain is one of the European countries with the lowest prevalence of major depressive episodes [5]. However, recent findings have shown an increased incidence of mental disorders and psychological distress in recent years in Spain [6,7,8]. As expected, psychotropic medication consumption has progressively increased in Spain, particularly among women [9,10,11]. However, it is important to note that different studies have suggested that mental disorders may not be the only factor or even the most important factor behind this increment [12,13]. Several works have reported a great difference between percentages of mental health diagnosis and psychotropic drug consumption [14]. This means that other clinical and nonclinical predisposing factors to psychotropic treatment should also be analyzed [11,14,15,16]. Interestingly, the European economic recession seems to have had a particular impact on mental healthcare, serving as a modulating and predisposing factor for mental disorders [17].

Several authors have highlighted the importance and utility of population health surveys for monitoring psychological morbidity and identifying potential associations between sociodemographic and/or health-related factors and psychiatric morbidity, allowing to identify groups at risk of developing mental illness [11,15,16,18,19]. The Spanish National Health Interview Survey (SNHIS) has been described as a useful instrument for the epidemiological investigation of common mental disorders such as self-reported depression and anxiety [19]. Interestingly, it can be also used to measure psychological distress based on the 12-Item General Health Questionnaire (GHQ-12) [20], which has been previously validated in Spanish and used on the general population and on populations with chronic diseases [21,22,23].

Using the SNHIS 2017, the aims of the present work were to (i) assess gender differences in the prevalence of self-reported mental disorders, psychological distress (GHQ-12 ≥ 3) and self-reported psychiatric medication consumption and to (ii) identify which sociodemographic and health-related variables are associated with reporting mental disorders, psychological distress (GHQ-12 ≥ 3) and consumption of psychotropic medications in the male and female populations.

## 2. Materials and Methods

### 2.1. Study Design and Study Population

This is an epidemiological cross-sectional study. The data for our investigation were obtained from the SNHIS 2017. Details on the methodology of the SNHIS 2017 are described elsewhere [24,25].

The SNHIS 2017 was designed to provide reliable estimates, at both national and regional levels, of the population living in Spain aged 15 years or over and included a total of 23,089 participants. However, in accordance with considerations of psychotropic drug prescription, we selected individuals aged ≥ 18 years, resulting in a study population of 22,141 subjects. The information collection period was from October 2016 to October 2017. Briefly, the SNHIS 2017 uses three-stage sampling, the first stage being the census tracts, the second the main family dwellings and the last stage involving the random selection (Kish method) of an adult (aged ≥ 15 years old) within each household [24]. The method used to collect the information is computer-assisted personal interview.

### 2.2. Study Variables

The variables included in the present study were selected based on questions related to the variables of sociodemographic characteristics health status and use of health services and to lifestyle behaviors. Details regarding the questions used to create our study variables can be found in Table S1 and in the SNHIS 2017 methodology description [24,25].

- Mental health status was measured using three dependent variables:
  1. The self-reported presence of a “mental disorder”, defined as the person interviewed reporting suffering from depression and/or anxiety, with these conditions having been diagnosed by a medical professional;
  2. The presence of “psychological distress”, assessed using the 12-item General Health Questionnaire 12 (GHQ-12). The Spanish version of the GHQ-12 has been validated, and a cutoff point of ≥3 is recommended to identify individuals with psychological distress [20,21,22,23];
  3. The variable “psychotropic drug consumption”, created using questions regarding the self-reported use of physician-prescribed medications in the last two weeks. We considered any of the following as psychiatric medications: “tranquilizers (anxiolytics)”, “sedatives (anxiolytics)”, “sleeping pills (anxiolytics)” and “antidepressants”.
- Independent variables included were classified into four types:

(i) sociodemographic characteristics: “gender”, “age groups”, “nationality”, “marital status”, “educational level” and “social class”; (ii) health status variables: “self-rated health” and self-reported presence of medical-professional-diagnosed concomitant chronic diseases (“hypertension”, “heart diseases”, “arthrosis”, “stroke”, “diabetes mellitus”, “malignant tumors”, “respiratory diseases“, “chronic pain” and “accident permanent injuries”); (iii) use of healthcare services in the last year (“emergency services”, “hospital admission”, “visit to physiotherapist” and “visit to psychologist”); and (iv) lifestyle variables (“obesity”, “alcohol consumption”, “current smoking habit” and “physical inactivity).

Detailed descriptions and categories for these variables are shown in Table S1.

### 2.3. Statistical Analysis

Qualitative variables were expressed as frequencies and percentages. Comparisons were carried out using Chi-squared test. If the participant answered, “Don’t know” or “Don’t answer”, they were excluded from the analysis of that variable. Multivariable analyses were performed using logistic regression, generating three models, one model for each dependent study variable. The models included variables with a significant association in the bivariate analysis or reported as relevant in the literature. Odds ratios (OR) with 95% confidence intervals (CI) are provided as measures of association. We considered as possible confounders of the multivariate logistic regression analysis the following variables: age, nationality, marital status, education level and social class. Statistical analysis was performed using the software SPSS 25.0. A *p* value < 0.05 was considered statistically significant (two tails).

### 2.4. Ethical Aspects

In accordance with the Spanish legislation, as we used a public access dataset with anonymous data, the approval of an ethics committee is waived. The database can be freely downloaded by anyone from the Spanish Ministry of Health webpage [26].

## 3. Results

### 3.1. Distribution of Characteristics of the Study Population

**Figure 1:**
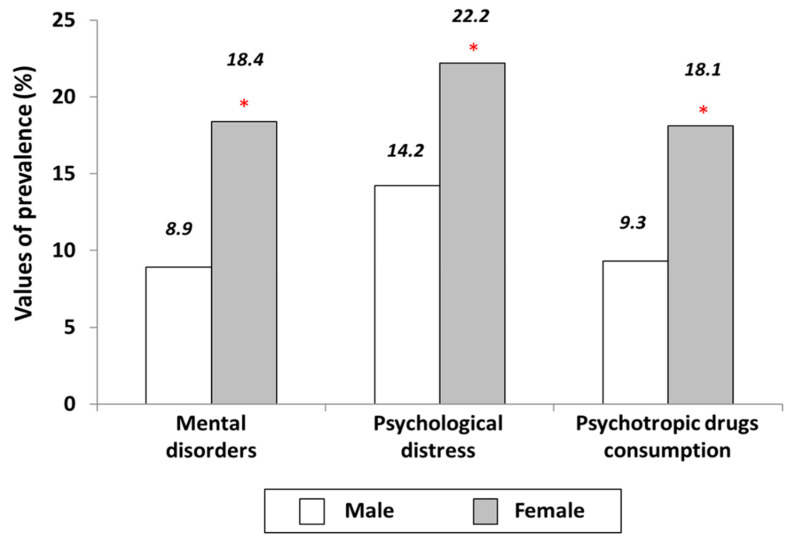
Prevalence of variables of mental disorders, psychological distress and psychotropic drug consumption according to gender. * *p* value < 0.001 for the comparison between men and women.

The study population included a total of 22,141 participants aged 18 years or over interviewed in the SNHIS 2017, which is considered a balanced population with respect to gender, with percentages of 48.6% and 51.4% for men (*n* = 10,751) and women (*n* = 11,390), respectively. As Figure 1 shows, crude prevalence of mental disorders (8.9% vs. 18.4%), psychological distress (14.2% vs. 22.2%) and psychotropic drug consumption (9.3% vs. 18.1%) was significantly higher among women compared to men. These results indicate a 2.07-fold higher crude prevalence for mental disorders, 1.56-fold for psychological distress and 1.95-fold for psychotropic medication consumption among women compared to men.

### 3.2. Prevalence of Mental Health Disorders, Psychiatric Distress and Psychotropic Drug Consumption According to Sociodemographic Variables. Comparison between Men and Women

In Table 1, the prevalence of mental disorder, psychological distress and psychotropic drug consumption is presented according to sociodemographic variables for each gender. Mental disorders, psychological distress and psychotropic drug consumption were significantly associated with the sociodemographic variables of age, nationality, marital status, education level and social class in the total population.

The highest prevalence of mental health disorders, psychiatric distress and psychotropic drug consumption was found in both genders for the categories of older than 67 years, not married, with primary studies and low social class.

Being a woman was significantly associated with a higher prevalence of suffering from mental health disorders, experiencing psychological distress and consuming psychiatric drugs than being a man, according to all categories of the sociodemographic variables included in the present study, with an exception made for the 18–37-year-old subgroup which did not reach significant differences for psychotropic drug consumption (*p* = 0.098).

### 3.3. Prevalence of Mental Health Disorders, Psychiatric Distress and Psychotropic Drug Consumption According to Health Status. Comparison between Men and Women

Table 2 shows the distribution of mental health disorders, psychiatric distress and psychotropic drug consumption prevalence according to different health status variables, including self-rated health by gender. According to the chronic conditions analyzed, the highest prevalence of mental disorders was found in women who suffered from concomitant stroke (43.3%), heart diseases (38.9%) and malignant tumors (38.1%). Meanwhile, the highest prevalence of psychologic distress and psychotropic drug consumption was found among women who suffered from mental disorders (53.6% and 63.6%, respectively), stroke (45.3% and 46.2%, respectively) and heart diseases (42.6% and 41.1%, respectively).

Being a woman was significantly associated with a higher prevalence of suffering from mental health disorders, experiencing psychiatric distress and consuming psychiatric drugs according to all categories of health status variables included in the present study. The only exception was the value of mental disorder prevalence, which did not reach statistical significance when a comparison was carried out among genders (*p* = 0.070).

### 3.4. Prevalence of Mental Health Disorders, Psychiatric Distress and Psychotropic Drug Consumption According to Use of Healthcare Services and Lifestyle-Related Variables. Comparison between Men and Women

All variables related to the use of healthcare services and lifestyle analyzed in the present work were significantly associated with mental health disorders, psychiatric distress and psychotropic drug consumption. As expected, it was found that women also showed a significantly higher risk of developing mental health disorders, experiencing psychological distress and consuming psychotropic medication compared to men according to lifestyle variables. In terms of visits to a psychologist, no significant differences were found in the prevalence of mental disorders, psychological distress or psychotropic medication consumption according to gender (Table 3).

### 3.5. Variables Associated to Mental Health Disorders, Psychiatric Distress and Psychotropic Drug Consumption after Multivariable Analysis

Table 4 shows the multivariable logistic regression adjusted ORs, identifying the potential predictors for mental health disorders, psychiatric distress and psychotropic drug consumption in the total study population.

After adjusting for possible confounders, women had a 1.74 (95% CI 1.54–1.96) fold probability of reporting mental disorders, 1.26 (95% CI 1.15–1.37) fold of reporting psychological distress and 1.26 (95% CI 1.15–1.45) fold of reporting psychotropic drug consumption compared to men (all *p* < 0.001).

Results revealed that in addition to being a woman, being aged 37–67 years; not being married; having a medium–low education level (secondary and primary studies); having poor self-rated health; visiting a psychologist; suffering from a chronic disease, such as stroke, malignant tumors, chronic pain and psychologic distress, or having permanent injuries caused by an accident; and having unhealthy lifestyles, including obesity, psychotropic drug consumption or a smoking habit were identified as risk factors for reporting mental disorders. On the other hand, being an immigrant and consuming alcohol were found to be protective factors against mental disorders among residents in Spain.

Our results also showed that positive predictors for psychological distress included not being married; coming from a low social class; having poor self-rated health; using emergency and psychological services or being admitted to hospital; suffering from a chronic disease, such as heart disease, stroke, diabetes mellitus, malignant tumors, respiratory diseases and chronic pain, or having permanent injuries caused by an accident; suffering from mental disorders; and having an unhealthy lifestyle, including a smoking habit and psychotropic drug consumption. By contrast, being older than 50 years and engaging in physical activity were described as negative predictors for psychological distress.

As Table 4 shows, positive predictors for psychotropic drug consumption were an age older than 37 years; not being married; low educational levels (primary and secondary studies); poor self-rated health; use of emergency and psychologic services and hospital admission; several chronic diseases, including hypertension, arthrosis, chronic pain, psychologic distress and mental disorders; and a smoking habit. Being an immigrant and consuming alcohol were found to be negative predictors for psychotropic drug consumption.

Table S2 shows the specific variables associated with the three study variables among men and women after multivariable analysis. Interestingly, there are several independent variables that have a different effect on mental health status and psychotropic drug consumption according to gender. Chronic diseases such as heart disease, stroke, permanent injuries and obesity were only identified as positive predictors for mental disorder among women. In addition, the variables of low social class and several chronic diseases (heart disease, arthrosis, malignant tumors and respiratory diseases) also showed differential effects on psychological distress risk according to gender. On the other hand, a nationality other than Spanish, primary level of education, several chronic diseases and alcohol consumption also seem to exert a different effect on psychotropic drug consumption between men and women.

## 4. Discussion

In the present study, the prevalence of mental disorder, psychologic stress and psychotropic treatment according to different sociodemographic, health- related and lifestyle variables was analyzed in a large and representative sample of the resident population in Spain based on data from the SNHIS 2017. Results emphasized the increased prevalence of mental disorders, psychological distress and psychotropic medication in women compared to men. In addition, sociodemographic, health status and lifestyle variables were identified as potential predictors for mental health status and psychotropic treatment.

According to the Institute for Health Metrics and Evaluation (IHME 2018), 17.3% of people across EU countries had a mental health problem in 2016, with an estimated prevalence of 18.3% for Spain [8]. This estimation is similar to results obtained in the present study, where percentages of mental disorder and psychological distress prevalence reached up to 13.8% and 18.3%, respectively. What is remarkable is that this prevalence is lower than the values of 15.4% for mental disorder and 22.1% for psychologic distress obtained in Spain using the previous SNHIS 2011–2012 [27]. At the European level, the highest prevalence of mental health disorders is located in Finland, the Netherlands, France and Ireland, with rates of 18.5% or more, while Romania, Bulgaria and Poland showed the lowest prevalence, with rates of less than 15% of the population. Therefore, according to SNHIS2017 data, Spain would be ranked among the European countries with the lowest prevalence of mental disorders [8]. These marked differences among European countries may be due to the fact that people living in countries with greater awareness and less stigma associated with mental illness, as well as easier access to mental health services, may be diagnosed more easily or may be more likely to self-report mental ill health. However, there are countries where mental disorders are still strongly stigmatized, and it is thought that it is better to simply avoid talking about mental illness [28]. Our results also showed a decreased prevalence of prescription of psychotropic drugs compared to those reported in previous studies using the SNHIS 2011–2012 (SNHIS2017: 13.9% vs. SNHIS2011-2012: 15.6%) [11]. This descending trend across time has also been observed in other studies conducted in Spain with other methodological designs [29,30]. It contrasts results obtained from several countries such as Norway, the USA or Canada, where an increased prescription of psychotropic drugs was reported during the last decade [31,32,33,34,35]. The decreased prevalence of prescription of psychotropic drugs observed in the present study may be explained, at least in part, by the particularly adverse economic crisis that affected Spain starting in 2008 [36], which only began to recede in 2015 [37], just before data recruitment started for the present study. In different countries, the adverse effects of economic recession on mental health have been described, and how mental health status has improved after the economic crisis receded [29,38,39,40]. Our results also revealed that crude and adjusted prevalence of mental disorders, psychological distress and psychotropic medication consumption were higher among women than among men. In accordance with our results, there is a large body of scientific evidence supporting the occurrence of poorer mental status health and increased psychotropic medication consumption among women compared to men [7,27,30,41,42,43]. Possible explanations for these results may be related to differences in emotional processing and coping skills, willingness to report diseases or structural inequalities and even intrinsic biological factors such as hormone mechanisms [29]. In addition, despite great changes in female gender roles in recent decades, traditional gender roles that entail housework, childbearing, gender discrimination and gender-based violence promote exposure to stressors that can lead to psychological distress among women [29].

Age was also identified as a positive predictor for mental disorder; however, only the age range of 37–67 years reached statistical significance. This finding is consistent with previous studies based on epidemiological surveys [7], suggesting that common mental disorders such as anxiety and depression have an early age of onset [44]. Age older than 50 years was also found to be a protector for psychologic distress. This observed pattern of decreased prevalence of mental disorder and psychologic distress with increasing age seems to be in accordance with the widely accepted socioemotional selectivity theory (SST) [45]. This theory concludes that older adults adopt a present-focused state of awareness, seeking the achievement of emotionally meaningful goals through relatives and friends, which increase the likelihood of experiencing positive emotions [45]. On the other hand, increased age was strongly identified as a predictor for psychotropic drug consumption. It is widely known that the prescription rate of psychotropic drugs increases with age, with mental health problems associated with aging identified as the main factor behind the use of these drugs [46,47]. However, our results suggest that mental health problems may not be the only factor or even the decisive one for psychotropic drug consumption, and therefore, the possibility of the presence of other predisposing factors must be considered. Regarding this last point, in addition to the presence of mental health problems, factors such as physiological alterations of sleep patterns, number of chronic disorders, limitations in functional abilities and even characteristics of those prescribing the medicine have been closely associated with the prescription of psychotropic drugs [48,49,50].

Additionally, in accordance with previous studies, not being married has been found to be a positive predictor for mental disease and psychologic distress. A plausible explanation for this may be that the married population may avoid feelings of loneliness and feel higher levels of emotional and psychological wellbeing [51,52].

Being an immigrant was found to be a negative predictor for poor mental health and psychotropic medication consumption. Different aspects closely related to immigration such as lack of social or economic factors or cultural changes have been widely associated with an adverse impact on mental health [53]. However, different conditions may modulate the relationship between immigration and high risk of developing mental alterations. A previous report performed in Spain concluded that immigrants who resided in Spain for less than 10 years showed a better state of mental health than the Spanish population [54]. Other works outside Spain have also reported that the mental health of immigrants was found to be similar or better than that of the native population of the destination country [55,56,57]. However, it is important to point out that data obtained about a good mental health state among immigrants may be biased, since people with poorer mental health would have lower possibilities to migrate. In this regard, different works have shown that physical and mental wellbeing were factors driving the decision to migrate [58,59].

The inverse relationship between mental health alterations and social class and education levels are two of the most well established in the field of mental health epidemiology [60,61]. However, our results did not reveal the abovementioned inverse relationship. Uniquely, secondary and primary levels of education were identified as a predictor for mental disorders, while education levels were found to predict neither psychological distress nor psychotropic drug consumption. These controversial results may be due to the effect of social class and educational level on mental health being modulated by other aspects closely associated with the welfare state of a country. In this regard, it is important to note that when the data for the present work were collected, Spain had Europe’s second highest unemployment rate, the third highest percentage of underemployed part-time workers who wished to work more (51%) and the second highest rate of youth unemployment (43%) [62]. With these data, it would be plausible to think that stress due to unemployment or temporary employment status would increase the risk of developing poor mental health, independently of social class and education level. However, when the effect of social class on psychological distress was analyzed according to gender, it was found that a low social class was a predictor of psychological distress among women. This may be associated with the demanding responsibilities of housework and parenting of children among women who belong to a low social class in Spain [63], which would provide additional stress in comparison to men, independently of employment status. 

Our results showed that the population with a negative perception of their health were in higher risk of mental disorders, psychologic distress and psychotropic drug consumption, agreeing with previous reports [11,19]. In addition, as was expected, psychologist consultations were also associated with poor mental health status and psychotropic medication consumption. Interestingly, our results also revealed that emergency and non-emergency hospital admission increased psychologic distress risk and psychotropic drug consumption. These findings may also be closely associated with the presence of chronic disorders. The diagnosis of chronic disorders often requires numerous visits to the doctor and exposes the patient to chronic stressors which may lead to psychological distress and, therefore, the prescription of psychotropic drugs. Regarding this point, we have found that chronic disorders such as malignant tumors, stroke, permanent injuries and pathologies with chronic pain are all characterized by a high emotional charge for patients due to treatment and psychosocial or labor stressors, and they were a positive predictor for poor mental health and psychotropic medication consumption. This is consistent with findings from previous works [11,64,65].

In line with other studies conducted in Spain and other countries, our analysis of variables associated with lifestyle revealed that obesity was a predictor of mental disorders [66,67,68,69]. Interestingly, our results also revealed that obesity was a predictor of mental disorders only for women. A negative self-perceived body image is closely associated with low levels of perceived social support, particularly in the female population [70,71]. Perceived social support plays a crucial role in enjoying a sense of general wellness and even as a stress-buffering process [72,73]. In our population, engaging in physical exercise was significantly associated with lower psychological distress. This protective effect of physical activity has been previously described in the Spanish general population [23]. Regarding the consumption of addictive substances, smoking was a predictor of mental disorders, psychologic distress and psychotropic drug consumption. There is strong evidence for an association between smoking and the development and progression of mental disorders and psychotropic drug consumption [11,74]. On the other hand, the negative association between consumption of alcohol and both mental disorders and psychotropic drug consumption found in our work may be because alcohol is used as an alternative substance instead of psychotropic medicines for those who are suffering from anxiety or depression, especially in the male population [75]. Accordingly, we found that the protective effect of alcohol consumption on psychotropic drug consumption was limited to the male population. 

As expected, a positive association was found between the presence of mental disorder, psychologic distress and psychotropic drug consumption. These variables are closely related to each other. The positive directional association between distress and mental disorder is widely accepted, indicating that any study claiming to focus on mental health should incorporate measures of distress and mental disorders [76].

The main strength of this work was the use of a representative sample of the population residing in Spain, which allowed quantifying the prevalence of poor mental health status and psychotropic drug consumption as well as identifying predictors involved in the processes mentioned above. However, there were also several limitations to this study. The SNHIS is based on self-reported data, and therefore, it may be affected by nonresponse bias, memory bias or the tendency of interviewees to give socially desirable responses. Moreover, national health surveys rely on household sampling, but we do not have data from institutionalized populations, nor from hospitals and prisons or marginalized groups (e.g., the homeless population), who often have high rates of mental disorders and psychotropic treatment [77]. These taken all together, it is possible that the prevalence of poor mental health status and psychotropic drug consumption may be underestimated. Finally, the use of a cross-sectional design means that causality cannot be inferred. 

## 5. Conclusions

In conclusion, our findings show a high prevalence of poor mental health status and psychotropic drug consumption in Spain, emphasizing that gender differences exist, with women being in higher risk of mental disorder, psychological distress and psychotropic drug consumption. However, this prevalence seems to have decreased in comparison to what was identified during analysis against the backdrop of a particularly hard economic crisis in Spain. Different sociodemographic, health status and lifestyle factors appear to be associated with a prevalence of mental health status and psychotropic medication consumption. Programs targeted at preventing, monitoring and controlling these gender differences in mental health problems should be implemented in primary care. Screening for these conditions should be considered, particularly in Spanish women, younger adults and individuals who are not married, have poor self-rated health, suffer from chronic diseases or have a smoking habit.

## Figures and Tables

**Table 1:** Prevalence of mental disorders, psychologic distress and psychiatric drug consumption among men and women according to sociodemographic variables. Results from the Spanish National Health Interview Survey 2017.

	Mental Disorders			Psychological Distress			Psychotropic Drug Consumption		
Variables	Men	Women	*p* Value	Men	Women	*p* Value	Men	Women	*p* Value
	*n* (%)	*n* (%)		*n* (%)	*n* (%)		*n* (%)	*n* (%)	
Age (years) ^a,b,c^									
18–37	154 (5.4)	254 (9.1)	<0.001	314 (11.1)	482 (17.5)	<0.001	107 (3.8)	131 (4.7)	0.098
38–49	232 (7.9)	416 (14.2)	<0.001	405 (14.0)	570 (19.6)	<0.001	220 (7.5)	319 (10.9)	<0.001
50–67	351 (11.1)	735 (22.6)	<0.001	488 (15.6)	754 (23.4)	<0.001	352 (11.2)	706 (21.7)	<0.001
>67	221 (12.0)	692 (28.6)	<0.001	308 (17.2)	686 (29.2)	<0.001	325 (17.7)	908 (37.5)	<0.001
Nationality ^a,b,c^									
Spanish	911 (9.6)	1932 (19.7)	<0.001	1392 (14.9)	2188 (22.6)	<0.001	948 (10.0)	1925 (19.6)	<0.001
Other	47 (3.6)	165 (10.6)	<0.001	123(9.2)	304 (19.8)	<0.001	56 (4.3)	139 (8.9)	<0.001
Marital status ^a,b,c^									
Married	530 (7.6)	1117 (16.9)	<0.001	900 (13.1)	1352 (20.6)	<0.001	652 (9.4)	1127 (17.0)	<0.001
Others	428 (11.2)	980 (20.6)	<0.001	615 (16.3)	1140 (24.5)	<0.001	352 (9.2)	937 (19.7)	<0.001
Education level ^a,b,c^									
Primary	346 (13.9)	877 (28.9)	<0.001	446 (18.3)	862 (29.1)	<0.001	378 (15.2)	963 (31.7)	<0.001
Secondary	487 (7.9)	913 (16.1)	<0.001	832 (13.5)	1177 (20.9)	<0.001	483 (7.8)	798 (14.1)	<0.001
University	95 (4.9)	216(8.9)	<0.001	208 (10.7)	357 (14.8)	<0.001	118 (6.1)	188(7.8)	0.032
Social Class ^a,b,c^									
Upper	117 (5.7)	238 (11.4)	<0.001	227 (11.2)	300 (14.4)	<0.001	145 (7.1)	230 (11.0)	<0.001
Middle	313 (8.5)	622 (17.5)	<0.001	494 (13.6)	735 (20.9)	<0.001	336 (9.2)	635 (17.8)	<0.001
Low	516 (10.5)	1157 (21.3)	<0.001	782 (16.1)	1382 (25.9)	<0.001	513 (10.4)	1112 (20.5)	<0.001

^a^ Significant association for mental disorders in the total population. ^b^ Significant association for psychologic distress in the total population. ^c^ Significant association for psychotropic drug consumption in the total population. *p*-Values represent comparison between the male and female population.

**Table 2:** Mental disorders, psychologic distress and psychiatric drug consumption according to health status variables. Results from the Spanish National Health Interview Survey 2017.

	Mental Disorders			Psychological Distress			Psychotropic Drug Consumption		
Variables	Men	Women	*p* Value	Men	Women	*p* Value	Men	Women	*p* Value
	*n* (%)	*n* (%)		*n* (%)	*n* (%)		*n* (%)	*n* (%)	
Self-rated health ^a,b,c^									
Very good, good	288 (3.6)	614 (8.2)	<0.001	602 (7.6)	854 (11.5)	<0.001	276 (3.5)	564 (7.6)	<0.001
Fair, poor, very poor	670 (23.9)	1483 (37.8)	<0.001	913 (33.3)	1637 (42.8)	<0.001	728 (25.9)	1500 (38.2)	<0.001
Hypertension ^a,b,c^	335 (13.1)	798 (29.9)	<0.001	484 (19.2)	821 (31.4)	<0.001	405 (15.9)	927 (34.8)	<0.001
Heart diseases ^a,b,c^	159 (15.9)	326 (38.9)	<0.001	233 (23.8)	346 (42.6)	<0.001	216 (21.6)	344 (41.1)	<0.001
Arthrosis ^a,b,c^	261 (19.0)	1010 (34.7)	<0.001	356 (26.4)	1016 (35.6)	<0.001	309 (22.5)	1142 (39.2)	<0.001
Permanent injuries ^a,b,c^	159 (18.2)	219 (37.4)	<0.001	238 (27.8)	243 (42.5)	<0.001	147 (16.8)	193 (32.9)	<0.001
Stroke ^a,b,c^	63 (27.9)	74 (43.3)	<0.001	74 (35.1)	72 (45.3)	0.040	81 (35.8)	79 (46.2)	0.040
Diabetes mellitus ^a,b^	145 (14.7)	300 (33.7)	<0.001	219 (22.6)	327 (37.8)	<0.001	180 (18.3)	348 (39.0)	0.040
Malignant Tumors ^a,b,c^	68 (17.4)	228 (38.1)	<.001	112 (29.5)	217 (37.5)	0.010	87 (22.3)	226 (37.7)	<0.001
Respiratory diseases ^a,b,c^	156 (16.7)	308 (29.1)	<0.001	239 (26.0)	358 (34.5)	<0.001	166 (12.7)	309 (29.2)	<0.001
Chronic pain ^a,b,c^	466 (18.1)	1413 (31.1)	<0.001	665 (26.0)	1521 (33.9)	<0.001	508 (19.7)	1375 (30.2)	<0.001
Mental disorders ^b,c^	958 (100)	2097 (100)	NA	505 (55.3)	1081 (53.6)	<0.001	578 (60.3)	1334 (63.6)	0.070
Psychological distress ^a,c^	505 (33.3)	1081 (43.4)	<0.001	1515 (100)	2492 (100)	NA	476 (31.4)	1028 (41.3)	<0.001

^a^ Significant association for mental disorders in the total population. ^b^ Significant association for psychologic distress in the total population. ^c^ Significant association for psychotropic drug consumption in the total population. *p*-Values represent comparison between the male and female population.

**Table 3:** Mental disorders, psychologic distress and psychiatric drug consumption according to use of healthcare services and lifestyle-related variables. Results from the Spanish National Health Interview Survey 2017.

	Mental Disorders			Psychological Distress			Psychotropic Drug Consumption		
Variables	Men	Women	*p* Value	Men	Women	*p* Value	Men	Women	*p* Value
	*n* (%)	*n* (%)		*n* (%)	*n* (%)		*n* (%)	*n* (%)	
Emergency services ^a,b,c^	381 (12.8)	956 (26.2)	<0.001	650 (22.3)	1147 (32.1)	<0.001	455 (15.3)	981 (26.9)	<0.001
Hospital admission ^a,b,c^	137 (15.8)	289 (31.2)	<0.001	260 (31.1)	366 (40.6)	<0.001	197 (22.8)	346 (37.2)	<0.001
Visit to physiotherapist ^a,b,c^	168 (10.0)	450 (21.2)	<0.001	296 (17.7)	518 (24.6)	<0.001	210 (12.4)	405 (19.1)	<0.001
Visit to psychologist ^a,b,c^	301 (70.3)	507 (69.5)	0.750	238 (58.0)	404 (57.0)	0.720	273 (63.9)	443 (60.6)	0.260
Psychotropic drug use ^a,b^	578 (57.7)	1334 (64.7)	<0.001	476 (49.1)	1028 (51.6)	0.210	1004 (100)	2064 (100)	NA
Obesity ^a,b,c^	208 (10.9)	508 (28.0)	<0.001	312 (16.5)	515 (28.9)	<0.001	208 (32.2)	475 (53.8)	<0.001
Alcohol consumption ^a,b,c^	581 (7.0)	937 (14.6)	<0.001	1065 (12.8)	1195 (18.7)	<0.001	618 (7.4)	872 (13.6)	<0.001
Smoking habit ^a,b,c^	344 (11.0)	480 (19.8)	<0.001	513 (16.6)	559 (23.1)	<0.001	300 (9.6)	386 (15.9)	<0.001
Physical inactivity ^a,b,c^	511 (7.2)	993 (15.1)	<0.001	744 (20.5)	1348 (28.7)	<0.001	549 (7.8)	923 (14.0)	<0.001

^a^ Significant association for mental disorders in the total population. ^b^ Significant association for psychologic distress in the total population. ^c^ Significant association for psychotropic drug consumption in the total population. *p*-Values represent comparison between the male and female population.

**Table 4:** Variables independently and significantly associated with mental disorders, psychologic distress and psychiatric drug consumption. Results from the Spanish National Health Interview Survey 2017.

Variables	Categories	Mental Disorders		Psychological Distress		Psychotropic Drug Consumption	
		OR (CI 95%)	*p* Value	OR (CI 95%)	*p* Value	OR (CI 95%)	*p* Value
Gender	Male	1		1		1	
	Female	1.74 (1.54–1.96)	<0.001	1.26 (1.15–1.37)	<0.001	1.29 (1.15–1.45)	<0.001
Age (years)	18–37	1		1		1	
	38–49	1.25 (1.05–1.50)	0.013	0.97 (0.86–1.10)	0.670	2.31 (1.87–2.85)	<0.001
	50–67	1.41 (1.17–1.70)	<0.001	0.78 (0.68–0.89)	<0.001	3.61 (2.92–4.46)	<0.001
	>67	0.87 (0.69–1.10)	0.247	0.57 (0.48–0.68)	<0.001	6.13 (4.79–7.83)	<0.001
Nationality	Spanish	1		1		1	
	Other	0.67 (0.55–0.82)	<0.001	0.96 (0.84–1.09)	0.514	0.77 (0.62–0.95)	0.013
Marital status	Married	1		1		1	
	Other	1.40 (1.25–1.58)	<0.001	1.25 (1.14–1.36)	<0.001	1.06 (0.94–1.19)	0.325
Level of education	University	1		1		1	
	Secondary	1.29 (1.07–1.56)	<0.001	0.88 (0.75–1.04)	0.126	1.24 (0.99–1.53)	0.051
	Primary	1.60 (1.31–2.02)	0.007	0.93 (0.82–1.06)	0.273	1.23 (1.02–1.48)	0.029
Social class	Upper	1		1		1	
	Middle	1.01 (0.84–1.22)	0.882	1.12 (0.97–1.27)	0.139	1.03 (0.86–1.23)	0.749
	Low	1.10 (0.91–1.33)	0.331	1.21 (1.06–1.39)	0.006	0.93 (0.77–1.12)	0.429
Self-rated health	Good	1		1		1	
	Poor	2.17 (1.91–1.33)	<0.001	2.92 (2.64–3.22)	<0.001	1.87 (1.64–2.12)	<0.001
Emergency services	Yes	0.95 (0.84–1.07)	0.399	1.31 (1.20–1.44)	<0.001	1.46 (1.30–1.65)	<0.001
Hospital admission	Yes	0.64 (0.53–0.77)	<0.001	1.26 (1.10–1.45)	0.001	1.29 (1.09–1.52)	0.003
Physiotherapist visit	Yes	0.91 (0.79–1.05)	0.219	0.98 (0.88–1.09)	0.707	1.08 (0.94–1.24)	0.294
Psychologist visit	Yes	7.50 (6.21–9.06)	<0.001	1.98 (1.68–2.32)	<0.001	5.10 (4.21–6.17)	<0.001
Hypertension	Yes	1.02 (0.88–1.16)	0.826	1.05 (0.94–1.17)	0.402	1.24 (1.09–1.41)	0.001
Heart diseases	Yes	1.13 (0.94–1.35)	0.185	1.22 (1.05–1.40)	0.007	1.07 (0.91–1.26)	0.411
Arthrosis	Yes	1.09 (0.94–1.26)	0.252	1.11 (0.99–1.25)	0.073	1.39 (1.22–1.59)	0.000
Permanent injuries	Yes	1.33 (1.10–1.61)	0.003	1.63 (1.42–1.88)	<0.001	0.87 (0.72–1.06)	0.166
Stroke	Yes	1.49 (1.06–2.09)	0.021	1.45 (1.11–1.91)	0.007	1.39 (1.02–1.89)	0.036
Diabetes mellitus	Yes	0.98 (0.82–1.18)	0.873	1.15 (1.00–1.33)	0.056	0.95 (0.80–1.12)	0.533
Malignant tumors	Yes	1.28 (1.03–1.58)	0.025	1.31 (1.10–1.56)	0.002	1.04 (0.85–1.28)	0.690
Respiratory diseases	Yes	1.03 (0.87–1.22)	0.732	1.26 (1.10–1.43)	<0.001	1.05 (0.90–1.24)	0.578
Chronic pain	Yes	1.72 (1.52–1.95)	<0.001	1.43 (1.30–1.57)	<0.001	1.42 (1.26–1.60)	<0.001
Obesity	Yes	1.23 (1.07–1.41)	0.004	0.93 (0.83–1.03	0.176	0.88 (0.76–1.00)	0.062
Alcohol consumption	Yes	0.85 (0.75–0.95)	0.007	0.93 (0.85–1.02)	0.126	0.87 (0.77–0.97)	0.017
Smoking habit	Yes	1.47 (1.29–1.68)	<0.001	1.13 (1.02–1.24)	0.017	1.17 (1.02–1.34)	0.021
Physical activity	Yes	0.99 (0.89–1.12)	0.959	0.68 (0.62–0.73)	<0.001	0.93 (0.83–1.04)	0.196
Psychotropic drugs	Yes	10.35 (9.15–11.69)	<0.001	1.77 (1.57–2.00)	<0.001	-	
Psychological distress	Yes	2.80 (2.48–3.15)	<0.001	-		1.81 (1.59–2.04)	<0.001
Mental disorders	Yes	-		2.72 (2.41–3.06)	<0.001	10.46 (9.26–11.82)	<0.001

Categories “yes” were compared to “no” (reference categories, not shown) for each variable. CI: confidence interval.

## Data Availability

This database can be downloaded freely and without cost from the website of the Ministry of Health, Social Services, and Equality (https://www.mscbs.gob.es/estadEstudios/estadisticas/encuestaNacional/encuesta2017.htm, accessed on 20 May 2021). All relevant data, however, are already presented within this paper.

## Supplementary Materials

The following are available online at https://www.mdpi.com/article/10.3390/ijerph18126350/s1, Table S1: Definition of dependent and independent variables used in our investigation according to the questions included in the Spanish National Health Interview Survey 2017 (SNHIS 2017). Table S2: Variables independently and significantly associated with mental disorders, psychologic distress and psychiatric drug consumption according to gender. Results from the Spanish National Health Survey 2017.
