# Supplementary material for: Gender Differences in the Prevalence of Mental Health, Psychological Distress and Psychotropic Medication Consumption in Spain: A Nationwide Population-Based Study
Source: Int J Environ Res Public Health. 2021 Jun 11;18(12):6350. doi: 10.3390/ijerph18126350 (PMC8296165; doi:10.3390/ijerph18126350)
Supplement: Supplementary file 1 [file ijerph-18-06350-s001.zip › ijerph-1212767-supplementary.pdf]

Supplementary Table S1. Definition of dependent and independent variables used in our investigation according to the questions included in the Spanish National Health Interview Survey 2017 (SNHIS 2017).

Questions	Description and answer	Variables	Categories
Have you suffered depression or anxiety over the previous 12 months? and "Has your medical doctor confirmed the diagnosis?"	Only those who answered yes to both question were considered as subject with mental disorders.	Mental disorders	1. Yes<br>2. No
12 items Global health questionnaire, (GHQ-12)[1, 2]*	The GHQ-12 consists of 12 items, each assessing the severity of a mental problem over the last few weeks using a 4-point Likert-type scale (from 0 to 3). Positive items were corrected from 0 (always) to 3 (never), and negative ones from 3 (always) to 0 (never). Scores on the GHQ-12 are obtained from a summation of responses to the 12 questions, with the first two response options scoring 0 and the last two scoring 1 (0-0-1-1).	Psychological distress GHQ12>3	Yes: GHQ12>3<br>No: GHQ12 ≤3
- Next I am going to read you a list of types of medications, please tell me which one or more of them have you taken in the last 2 weeks?<br>- Was it prescribed by the doctor?	A list of 23 medications are read the person interviewed. For those medications with an affirmative answer in the first question, the second are completed consecutively for each specific medication.<br>1. Tranquilizers sedatives or "sleeping pills",<br>2. Antidepressants	Consumption of psychiatric medications	Yes: Those who answered "yes" to both question for options 1 and/or 2<br>No: Answered "no" to one or both questions for options 1 and 2
Which is your sex?	Men<br>Women	Gender	Male<br>Female
How old are you?	Age in years	Age groups	18-37 years<br>38-49 years<br>50-67 years<br>68 or more
What is your country of birth?	1. Spain<br>2. Foreign	Nationality	1. Spanish<br>2. Foreign
What is your legal marital status?	1. Single<br>2. Married<br>3. Widower<br>4. Legally separated<br>5. Divorced	Marital status	Married: Option 1.<br>Other: Options 1 and 3 to5
What level of education have you completed?	1. Does not know how to read or write<br>2. Incomplete primary education<br>3. Complete primary education (<br>4. First stage of Secondary Education, with or without a qualification<br>5. Elementary Spanish Upper Secondary Education])<br>6. Upper secondary education<br>7. Intermediate vocational training or equivalent<br>8. Advanced vocational training or equivalent<br>9. University studies or equivalent<br>10. Over university (master, PhD….)	Educational level	Primary Options 1 to 3<br>Secondary Options 4 to 8<br>University Options 9 and 10
The social class categories have been taken from the proposal made by the Spanish Society of Epidemiology's	National Classification of Occupations 2011 (CNO2011), [4]	Social Class	Upper<br>Meddle

(SEE) Working Group on Determinants, in which social class is assigned according to occupation[3]			Lower
In the past twelve month. How is your perception of your general health status?	1. Very good<br>2. Good,<br>3. Fair<br>4. Bad<br>5. Very bad.	Self-rated health	Very good/ good: Options 1 and 2<br>Fair/poor/very poor: Options 3 to5
1.Do you have or have you ever had any of the following diseases or heath conditions?<br>2. Have you suffered this disease/health condition in the past 12 months?<br>3. Was this disease/health condition been diagnosed by a doctor?	A card with a list of 32 conditions is shown to the person interviewed after the first question, and for those conditions reported by the participant, the second and third questions are completed consecutively for each specific condition.	• Hypertension<br>• Heart diseases (myocardial infarction, angina, coronary disease)<br>• Arthrosis<br>• Permanent injuries<br>• Stroke<br>• Diabetes mellitus<br>• Malignant tumours<br>• Respiratory diseases (asthma, chronic obstructive pulmonary disease)<br>• Chronic pain (neck pain and/or low back pain)<br>Accident with permanent injuries	Yes: Answered affirmative to the three questions.<br>No: All the rest
In the last 12 months, have you had to use an emergency service for any problem or illness?	1. Yes<br>2. No	Emergency services	1. Yes<br>2. No
In the last 12 months, have you had to be hospitalized for at least one night?	1. Yes<br>2. No	Hospital admission	1. Yes<br>2. No
In the last 12 months, have you visited a physiotherapist?	1. Yes<br>2. No	Visit to physiotherapist	1. Yes<br>2. No
In the last 12 months, have you visited a psychologist?	1. Yes<br>2. No	Visit to psychologist	1. Yes<br>2. No
1. Could you tell me how tall you are, approximately, without shoes?<br>2. Could you tell me your weight, approximately, without shoes and clothes?	Body mass index is calculated with the formulae:<br>Weight in Kg/ (Height in meters)^2	Obesity	Yes: If Body mass index is 30 or over.<br>No: Yes: If Body mass index is under 30
During the past 12 months, how often have you had alcoholic beverages of any kind (i.e. beer, wine, spirits, distilled and mixed drinks, or other alcoholic beverages)	1. Daily or almost daily<br>2. 5-6 days per week<br>3. 3-4 days per week<br>4. 1-2 days per week<br>5. 2-3 days in a month<br>6. Once a month<br>7. Less than once a month<br>8. Not in the last 12 months, have I stopped drinking<br>9. Never or just a few sips to taste it throughout life	Alcohol consumption in last 12 months	Yes. Options 1 to 6<br>No: Option 7 to 9
Could you tell me if you smoke?	1. Yes, I smoke daily<br>2. Yes, I smoke, but not daily	Current smoking habit	Yes. Options 1 and 2<br>No: Option 3 and 4

	3. I don't currently smoke but have smoked before<br>4. I neither smoke nor have I ever smoked regularly ..		
Which of these possibilities best describes how often you do some physical activity in your free time?	1. I don't exercise. I occupy my free time almost completely sedentary.<br>2. I do some occasional physical or sports activity.<br>3. I do physical activity several times a month<br>4. I do sports or physical training several times a week	Physical inactivity	Yes. Option 1<br>No: Option 2 to 4

[1] D. Goldberg, P. Williams, User's Guide to the General Health Questionnaire, NFER-Nelson, Berkshire, 1988.[2] Muñóz PE, et al. Adaptación española del General Health Questionnaire (G.H.Q.) de D. P. Goldberg (un método de identificación de casos psiquiátricos en la comunidad of D. P. Goldberg.*Arch Neurobiol (Madr)*. 1979;42(2):139-158. [3]Instituto Nacional de Estadistica. Encuesta Nacional de Salud 2017. [National Health Survey 2017]. Available at https://www.ine.es/daco/daco42/clasificaciones/cno11_notas.pdfhttps://www.ine.es/en/metodologia/t15/t153041917_en.pdf. Accessed 12 February 2021. [4]Instituto Nacional de Estadistica. National Classification of Occupations 2011 (CNO2011), Available at https://www.ine.es/daco/daco42/clasificaciones/cno11_notas.pdf Accessed 26 February 202

Supplementary Table S2. Variables independently and significantly associated with mental disorders, psychologic distress and psychiatric drugs consumption according to gender. Results from the Spanish National Health Survey 2017

**VARIABLES**	**CATEGORIES**	**MENTAL DISORDERS** OR (CI 95%)				**PSYCHOLOGICAL DISTRESS** OR (CI 95%)				**PSYCHOTROPIC DRUGS CONSUMPTION** OR (CI 95%)			
		MALE	***P value***	***FEMALE***	***P value***	***MALE***	***P value***	***FEMALE***	***P value***	***MALE***	***P value***	***FEMALE***	***P value***
**Age (years)**	*18-37*	1		1		1		1		1		1	
	38-49	1.33 (0.98-1.82)	.066	1.23 (0.99-1.53)	.061	1.03 (0.85-1.25)	.713	0.93 (0.79-1.09)	.415	2.12 (1.52-2.95)	<.001	2.43 (1.85-3.19)	<.001
	50-67	1.61 (1.16-2.23)	.004	1.35 (1.07-1.70)	.011	0.85 (0.68-1.05)	.131	0.74 (0.62-0.88)	.001	2.52 (1.79-3.56)	<.001	4.49 (3.41-5.91)	<.001
	>67	1.01 (0.67-1.51)	.936	0.84 (0.62-1.12)	.245	0.68 (0.52-0.90)	.007	0.51 (0.40-0.65)	<.001	4.17 (2.81-6.18)	<.001	7.96 (5.98-10.96)	<.001
**Nationality**	*Spanish*	1		1		1		1		1		1	
	Foreign	0.53 (0.35-0.78)	.002	0.73 (0.58-0.92)	.009	0.81 (0.65-1.81)	.063	1.06 (0.90-1.24)	.474	0.83 (0.58-1.19)	.323	0.77 (0.60-0.99)	.045
**Marital status**	*Married*	1		1		1		1		1		1	
	Others	1.81 (1.47-2.26)	<.001	1.24 (1.07-1.43)	.003	1.35 (1.16-1.56)	<.001	1.19 (1.06-1.34)	.002	1.00 (0.81-1.24)	.932	1.02 (0.88-1.18)	.736
**Level of education**	*University*	1		1		1		1		1		1	
	Secondary	1.44 (0.98-2.12)	.058	1.71 (1.31-2.24)	<.001	0.89 (0.69-1.15)	.384	0.87 (0.71-1.07)	.204	1.06 (0.74-1.51)	.724	1.31 (1.00-1.72)	.049
	Primary	1.05 (0.75-1.47)	.742	1.42 (1.13-1.78)	.002	0.88 (0.71-1.09)	.263	0.96 (0.81-1.13)	.639	1.04 (0.76-1.42)	.778	1.32 (1.04-1.67)	.019
**Social Class**	*Upper*	1		1		1		1		1		1	
	Middle	1.08 (0.78-1.51)	.622	1.08 (0.78-1.51)	.913	1.04 (0.84-1.28)	.707	1.16 (0.97-1.39)	.092	1.00 (0.74-1.36)	.955	1.05 (0.83-1.32)	.651
	Low	1.13 (0.81-1.59)	.462	1.13 (0.81-1.59)	.504	1.11 (0.89-1.38)	.346	1.28 (1.07-1.54)	.006	0.89 (0.65-1.22)	.480	0.97 (0.77-1.23)	.834
**Self-rated health**	*Good*	1		1		1		1		1		1	
	Poor	2.43 (1.94-3.06)	<.001	2.03 (1.73-2.39)	<.001	2.79 (2.39-3.26)	<.001	2.96 (2.60-3.36)	<.001	2.28 (1.85-2.81)	<.001	1.66 (1.41-1.95)	<.001

**Emergency services**	*Yes*	0.92 (0.75-1.14)	.487	0.93 (0.83-1.12)	.720	1.37 (1.19-1.58)	<.001	1.27 (1.13-1.43)	<.001	1.48 (1.22-1.81)	<.001	1.46 (1.26-1.70)	<.001
**Hospital admission**	*Yes*	0.55 (0.40-0.76)	<.001	0.67 (0.53-0.85)	.001	1.47 (1.20-1.80)	<.001	1.10 (0.91-1.32)	.298	1.43 (1.10-1.86)	.007	1.22 (0.98-1.22)	.075
**Visit physiotherapist**	*Yes*	0.72 (0.55-0.95)	.020	1.00 (0.84-1.19)	.948	1.14 (0.97-1.36)	.109	0.90 (0.78-1.03)	.140	1.43 (1.13-1.81)	.003	0.92 (0.77-1.10)	.382
**Visit psychologist**	*Yes*	9.66 (7.01-13.31)	<.001	6.21 (4.91-7.89)	<.001	2.14 (1.64-2.81)	<.001	1.85 (1.51-2.27)	<.001	6.18 (4.49-8.51)	<.001	4.49 (3.52-5.71)	<.001
**Hypertension**	*Yes*	1.14 (0.90-1.43)	.272	0.97 (0.82-1.16)	.794	1.02 (0.86-1.20)	.795	1.06 (0.91-1.22)	.420	1.06 (0.86-1.31)	.562	1.34 (1.14-1.58)	<.001
**Heart diseases**	*Yes*	0.83 (0.61-1.12)	.226	1.33 (1.05-1.67)	.015	1.07 (0.87-1.32)	.488	1.36 (1.11-1.65)	.002	1.32 (1.03-1.70)	.025	0.93 (0.74-1.16)	.525
**Artrosis**	*Yes*	1.12 (0.86-1.45)	.372	1.11 (0.93-1.32)	.245	1.06 (0.87-1.28)	.534	1.18 (1.02-1.38)	.027	1.25 (0.99-1.57)	.058	1.39 (1.22-1.59)	<.001
**Permanent injuries**	*Yes*	1.20 (0.90-1.61)	.204	1.46 (1.13-1.89)	.003	1.53 (1.26-1.87)	<.001	1.74 (1.41-2.16)	<.001	0.82 (0.62-1.10)	.194	0.93 (0.71-1.21)	.608
**Stroke**	*Yes*	1.15 (0.70-1.88)	.559	1.73 (1.07-2.81)	.025	1.40 (0.96-2.02)	.073	1.51 (0.99-2.29)	.051	1.82 (1.21-2.73)	.004	0.95 (0.59-1.53)	.847
**Diabetes mellitus**	*Yes*	0.98 (0.73-1.32)	.916	0.98 (0.77-1.24)	.906	1.13 (0.91-1.40)	.252	1.16 (0.95-1.42)	.139	1.01 (0.77-1.31)	.923	0.93 (0.74-1.17)	.555
**Malignant Tumors**	*Yes*	0.97 (0.65-1.46)	.902	1.42 (1.10-1.84)	.007	1.48 (1.12-1.96)	.006	1.23 (0.99-1.54)	.060	1.11 (0.78-1.58)	.544	1.00 (0.78-1.30)	.949
**Respiratory diseases**	*Yes*	1.17 (0.89-1.55)	.249	0.92 (0.74-1.14)	.480	1.34 (1.10-1.63)	.003	1.17 (0.98-1.38)	.071	1.02 (0.78-1.32)	.873	1.06 (0.85-1.31)	.576
**Chronic pain**	*Yes*	1.52 (1.23-1.88)	<.001	1.81 (1.56-2.11)	<.001	1.45 (1.25-1.68)	<.001	1.41 (1.25-1.60)	<.001	1.53 (1.26-1.87)	<.001	1.39 (1.19-1.62)	<.001
**Obesity**	*Yes*	1.05 (0.83-1.34)	.646	1.34 (1.13-1.59)	.001	0.94 (0.80-1.11)	.505	0.91 (0.78-1.05)	.200	0.93 (0.75-1.17)	.583	0.86 (0.72-1.03)	.108
**Alcohol consumption**	*Yes*	0.78 (0.62-0.97)	.030	0.89 (0.77-1.02)	.117	1.00 (0.76-1.17)	.952	0.89 (0.79-1.00)	.057	0.74 (0.61-0.91)	.005	0.99 (0.785-1.15)	.924

**Smoking habit**	*Yes*	1.44 (1.16-1.79)	.001	1.46 (1.24-1.73)	<.001	1.15 (1.00-1.33)	.048	1.11 (0.97-1.27)	.118	1.14 (0.92-1.41)	.209	1.19 (0.99-1.42)	.051
**Physical activity**	*Yes*	1.08 (0.88-1.32)	.447	0.95 (0.83-1.12)	.547	0.61 (0.53-0.69)	<.001	0.72 (0.64-0.81)	<.001	1.10 (0.91-1.33)	.318	0.84 (0.73-0.97)	<.001
**Psychotropic drugs**	*Yes*	13.28 (10.71-16.46)	<.001	9.15 (7.89-10.65)	<.001	1.77 (1.57-2.00)	<.001	1.77 (1.52-2.00)	<.001	--		--	
**Psychological distress**	*Yes*	3.56 (2.90-4.38)	<.001	2.46 (2.12-2.85)	<.001	--		--		1.78 (1.44-2.19)	<.001	1.97 (1.53-2.09)	<.001
**Mental disorders**	*Yes*	--		--		3.41 (2.78-4.18)	<.001	2.40 (2.08-2.78)	<.001	13.51 (10.92-16.71)	<.001	9.24 (7.94-10.74)	<.001

Categories *"yes"* were compared to *"no"* for each variable. CI: confidence interval
